# Comparative transcriptome analysis among parental inbred and crosses reveals the role of dominance gene expression in heterosis in *Drosophila melanogaster*

**DOI:** 10.1038/srep21124

**Published:** 2016-03-01

**Authors:** Xianwen Wu, Rongni Li, Qianqian Li, Haigang Bao, Changxin Wu

**Affiliations:** 1 College of Animal Science and Technology, China Agricultural University, Beijing, China; 2 National Engineering Laboratory for Animal Breeding, China Agricultural University, Beijing, China

## Abstract

We observed heteroses for body weight in *Drosophila melanogaster* after generating hybrids from three inbred lines. To better understand the mechanism for this phenomenon at the mRNA level, we compared the mRNA profiles of the parental and hybrid lines using high-throughput RNA-seq. A total of 5877 differentially expressed genes (DEGs) were found and about 92% of these exhibited parental expression level dominance. Genes in the dominance category were functionally characterized using the Kyoto Encyclopedia of Genes and Genomes (KEGG) and the gene classifications offered by the Gene Ontology (GO) Consortium. The analysis identified genes associated with crucial processes such as development and growth in all three crosses. Functional assignments involving aminoglycan metabolism, starch and sucrose metabolism, and galactose metabolism are significantly overrepresented amongst the 215 common dominance DEGs. We conclude that dominance DEGs are important in heteroses in *Drosophila melanogaster* and contribute specifically to body weight heterosis.

Heterosis, or hybrid vigor, refers to the phenotypic superiority of an F_1_ hybrid over its parents, observed and exploited in many crops and livestock^1^,^2^,^3^,^4^,^5^. Heterosis has been known for centuries and is widely used to improve crop and animal production. However, the biological mechanisms underlying the phenomenon are not well understood^6^. Hundreds of investigations have been performed to uncover the genetic and molecular mechanism of heterosis, using various methods and with diverse results^7^. For example, Krieger *et al.* (2010) reported that hybrid vigor in tomato production could be caused by a single heterozygous gene^8^. In contrast, abundant evidence suggests that the heterosis relevant to most economically interesting traits is controlled by large numbers of genes rather than individual loci^9^,^10^,^11^.

Dominance and overdominance are the two hypotheses that have been proposed to explain heterosis, but they were not originally expressed in terms of molecular mechanisms^12^. In the dominance hypothesis, heterosis occurs when less desirable alleles from one parent are suppressed by more desirable alleles from the other parent. In the overdominance hypothesis heterosis occurs when two parental alleles, acting together, result in a superior trait. More recently, both hypotheses have been reinterpreted in terms of additivity and nonadditivity when used to categorize differences in gene expression between hybrids and parents that may be important in heterosis^13^,^14^. In additive expression, a gene in a hybrid is expressed at a level equal to the mean of the values observed in the parents, while in nonadditive expression, the observed levels differ from the parental mean. Swanson-Wagner RA (2006) further categorized nonadditive gene expression into high parent dominance, low parent dominance, underdominance and overdominance^14^. Additive gene expression may also be associated with heterosis, though this is rarely observed^15^.

Recently, high-throughput sequencing technologies have been used to analyze the relationship between heterosis and gene expression differences between hybrids and their parents. After conducting mRNA and small RNA transcriptome analyses, Li *et al.* (2014) found that most protein coding genes exhibited a parental dominance pattern in their expression levels in nascent hexaploid wheat and contributed to heterosis^13^. Using digital gene expression profiling, Ding *et al.* (2014) showed that the genes associated with heterosis in early maize ear inflorescence development exhibited dominance and overdominance expression patterns^16^. Wang *et al.* (2015) reported that overdominance expression plays an important role in silkworm heterosis, though other gene expression patterns also affect silkworm heterosis^17^.

*Drosophila melanogaster* is one of the most widely used classical model organisms to study inheritance, development, evolution and myriad other phenomena^18^,^19^,^20^. However, except for a study in silkworm^17^, animal heterosis has not been examined using RNA-seq analyses. In our study, we examined heterosis in *Drosophila melanogaster* by performing RNA-seq analyses in three F1 hybrids and their parental inbred lines. Focusing on the body-weight trait, we predicted that combinations between parental lines differing in body weight would exhibit heterosis. Our data suggests that parental expression level dominance plays an important role in heterosis in *Drosophila melanogaster*. Given the similarities between *Drosophila melanogaster* and other organisms^21^,^22^, our results provide a baseline for comparison with studies of heteroses in agricultural animals.

## Results

### Heterosis in three F1 hybrid lines

WT (wild-type) flies and two additional parental inbred lines, differing in body weight, were used to generate F1 hybrids. Flies from the eyw line (characterized by ebony-body, yellow-body and white-eye) are typically lighter than WT flies, while flies from the w1118 line (characterized by white-eye) are typically heavier than WT. Body weights of the parental lines and three F1 hybrids (♂WT × ♀eyw, ♂w1118 × ♀WT, and ♂eyw × ♀WT) are shown in Table 1, along with the mean parental value (MPV) and rate of heterosis (RH) for each cross. Heterosis is apparent in all crosses, with the maximum heterosis rate observed Cross 3.

### Evaluation of RNA sequencing data quality

To analyze the gene expression profiles of the F1 hybrids and their parents, two cDNA libraries were constructed for each strain and subjected to sequencing using the Illumina HiSeq 2000 platform. Over 55 million short reads of raw data were generated for each strain, and more than 49 million high quality reads (Q-score > 20 for ≥ 94% of nucleotides in each read) were selected for further analyses. Approximately 82% of the high quality reads in each sample mapped to the reference genome. 98% of reads could be assigned to exonic regions, 0.7~1.1% to intronic regions and 0.6~0.8% to intergenic regions (Table S1). The correlation coefficients for the two biological replicates from each cross and its parents ranged from 0.964–0.993 (Fig. S1).

### Divergence of expression between F1 hybrid and parental inbred lines

**Figure 1:**
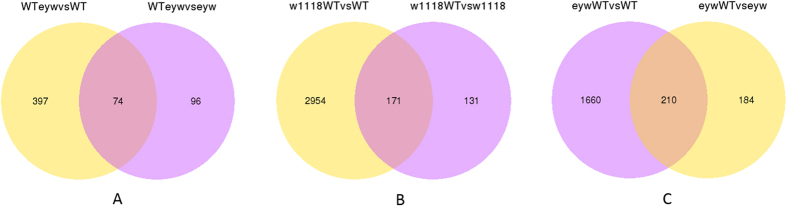
Differential expression of genes between F1 hybrids and parental inbred lines. Gene expression values were normalized as RPKM prior to comparing expression levels. Diagram (**A**) WT × eyw vs WT shows the number of differentially expressed genes in a comparison of the WT × eyw hybrid with the WT parent; WT × eyw vs eyw shows the number of differentially expressed genes in a comparison of the WT × eyw hybrid with the eyw parent; the overlapping region shows the number of differentially expressed genes that are in common. The diagrams for crosses (**B**) and (**C**) are arranged similarly. The total number of DGEs is: A (567) + B (3256) + C (2054) = 5877.

Of 16051 genes, 5877 DEGs (differentially expressed genes) were identified after comparing gene expression profiles of the F1 hybrids and their parents (Fig. 1). Eight genes were set aside because in some samples they had no associated reads, yielding 5869 DEGs for further analysis. Most DEGs were observed in comparisons between the WT line and its hybrids (left circles in Fig. 1A–C). The proportions were 91%, 83%, and 73% in the crosses of ♂w1118 × ♀WT, ♂eyw × ♀WT, and ♂WT × ♀eyw, respectively.

**Figure 2:**
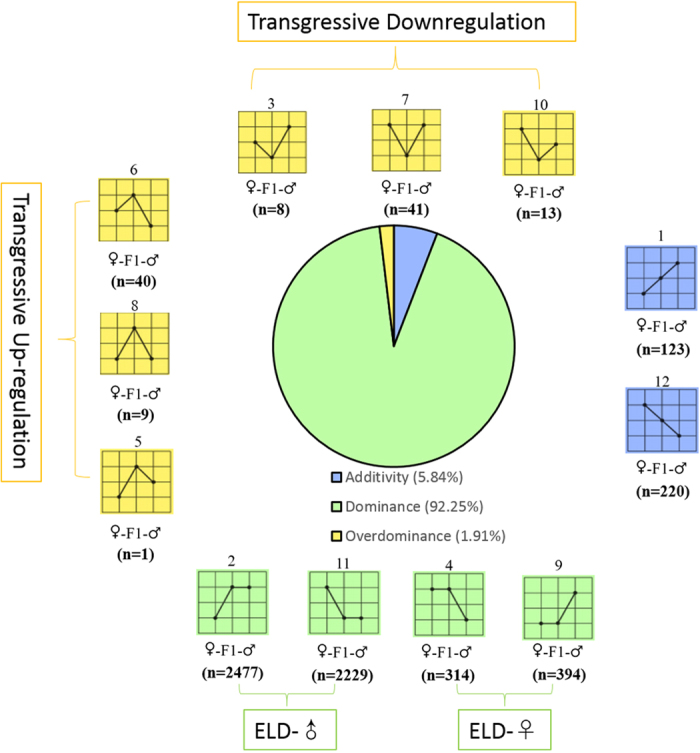
Classification of differentially expressed genes. Genes were classified according to the level of expression exhibited by parental and hybrid lines. Classes 1 and 12 (blue) are additive expression genes; Classes 2, 11, 4 and 9 (green) are dominance expression genes; Classes 5, 6, 8, 3, 7 and 10 (yellow) are overdominance expression genes. The overdominance class is subdivided into genes exhibiting transgressive upregulation (left) and transgressive downregulation (top). Each class is accompanied by diagrams representing the relative expression levels observed in the maternal parent (left point), F1 (middle point), and paternal parent (right point). The total number of differentially expressed genes in each class is shown as n.

All DEGs can be grouped into one of 12 expression classes (Fig. 2), following the conventions of Rapp *et al.*^23^ and Li *et al.*^24^. Relative to parental values, DEG expression levels in the F1 lines displayed additivity (Class 1 and 12), dominance (Class 2, 11, 4, and 9) or overdominance (Class 3, 7, 10, 6, 8, and 5). The dominance classes can be subdivided into expression-level dominance (ELD) for the paternal parent (ELD-♂, Class 2 and 11) and expression-level dominance for the maternal parent (ELD-♀, Class 4 and 9). The classification results for all three crosses are shown in Table S2. About 6% of DEGs displayed additive expression in F1 hybrids relative to their parental inbred lines, and 1.91% exhibited overdominance, either as transgressive down-regulation (1.06%) or transgressive up-regulation (0.85%) (Fig. 2). About 90% of DEGs in each cross displayed dominance expression (Fig. 2; Table S2). Furthermore, over 90% of DEGs in this category exhibit expression patterns consistent with the WT parent (♂w1118 × ♀WT and ♂eyw × ♀WT in category ELD-♂, and ♂WT × ♀eyw in category ELD-♀).

Correlations between expression patterns among the hybrids and their parents were investigated using hierarchical cluster analysis (Genecluster 3.0). The expression pattern of the w1118 strain was clearly distinct from WT and eyw, while there were small differences between the WT and eyw strains (Fig. S2). The expression pattern in one F1 hybrid (eywWT) was more similar to the pattern observed in its paternal parent than its maternal parent. Finally, patterns in the other two hybrids were similar, but were quite different from their parents (Fig. S2).

### Functional analysis of dominance expression genes

The GOseq R package (Release 2.12)^25^ was used for the functional classification of dominance DEGs from each cross. 5172 DEGs were assigned to 48 functional subcategories. Nearly all GO terms (41 of 48) were represented identically in all the three crosses (Fig. S3). As shown in Fig. S3, most DEGs were associated with biological processes (metabolic process, cellular process, response to stimulus, multicellular organismal process, developmental process, localization and biological regulation; cellular component of cell, cell part and organelle; and molecular functions in catalytic activity and binding). Several unique DEGs were associated with cell killing and viral reproduction, cellular component of virion and virion part, or molecular functions in auxiliary transport protein activity and nutrient reservoir activity. Some DEGs in Cross 2 and 3, but not in Cross 1, were associated with rhythmic process.

The KOBAS (v2.0)^26^ application was used to associate DEGs with metabolic pathways within the KEGG pathway database. As shown in Fig. S4, 57 pathways were overrepresented; of them, 13 were significantly overrepresented in Cross 1 (Qvalue < 0.064), 14 overrepresented in Cross 2, and 6 overrepresented in Cross 3. Two important pathways implicated in drug metabolism, cytochrome P450 and metabolism of xenobiotics by cytochrome P450, were significantly enriched in the three crosses. Two pathways (galactose metabolism, and starch and sucrose metabolism) were significantly overrepresented in Crosses 1 and 2, and a glutathione metabolism pathway was significantly overrepresented in Crosses 2 and 3.

Two hundred and fifteen dominance DEGs were identified as common to all crosses (Fig. S5; Table S3). GO and KEGG analyses revealed that two pathways (starch and sucrose metabolism, and galactose metabolism) were significantly overrepresented (corrected P-value < 0.01), involving eight genes (Fig. S6; Table S4; Table 2). The aminoglycan metabolic process was also significantly overrepresented (corrected P-value < 0.05), involving 10 genes (Fig. S7; Table S5; Table 2). Of these 18 genes, all but one (FBgn0035430) were in classes 9 or 11.

### qPCR validation of dominance differentially expressed genes

Ten of the 18 genes in Table 2 were selected for quantitative real time PCR analysis. Gene names, IDs, primer sequences, product lengths and Tm values are shown in Table S6. The results of qPCR validation are shown in Fig. S8. The data in Fig. S8 and Table 2 confirm that the expression patterns of these genes are consistent with RNA-Seq analysis and their classification.

## Discussion

Heterosis is widely applied in agriculture, but its molecular genetic mechanism remains a contentious subject. Uzarowska A *et al.* (2007)^27^ and Guo M *et al.* (2006)^28^ reported that divergent patterns of expression between F1 hybrids and their parents play a significant role in hybrid vigor. To examine this more closely, we used three inbred lines of *Drosophila melanogaster* to construct three F1 hybrids, and then generated transcriptome profiles for each hybrid and parental line using high-throughput mRNA sequencing technology. More than 5000 DEGs were selected and classified, revealing that 92% of DEGs exhibited a dominant expression pattern while just 6% and 1.91% of DEGs exhibited additive and overdominance gene expression patterns, respectively. Heteroses for growth, immunity, reproduction, or other phenomena are observed in hybrids from crosses of different strains of the same animal. Since the vast majority of DEGs in our experiment exhibited dominance, we suggest that dominance gene expression plays an important role in *Drosophila melanogaster* heteroses, although we cannot exclude effects due to additive and overdominance expression patterns.

We observed heterosis for body weight in all three crosses (Table 1). 215 common DEGs were found by expression profile analysis and at least 74 of these were potentially related to body weight, such as *Ubiquitin specific*^29^, *dDcp1* (Decapping protein 1)^30^,^31^ and others (Table S1). We speculate that these DEGs play important roles in heterosis for body weight. The 215 common DEGs were subjected to GO and KEGG pathway enrichment analyses. Genes associated with one GO term (aminoglycan metabolic process) and with two KEGG pathways (starch and sucrose metabolism, and galactose metabolism) were over-represented, consistent with a close relationship between carbohydrate metabolism and body weight.

A total of 18 genes were associated with the three process/pathways (Table 2). Their functional annotations suggest some of the mechanisms involved in heterosis for body-weight. *tobi* (target of brain insulin), *Mal-A2* (Maltase A2), *Mal-A1* (Maltase A1), *Mal-A8* (Maltase A8), and *Mal-A7* (Maltase A7) are all orthologs of alpha-glucosidase which hydrolyze starch molecules to linear malto-oligosaccharides in *Drosophila melanogaster*^32^. Inhibition of alpha-glucosidase activity can delay carbohydrate absorption and reduce bodyweight in humans^33^. Referring to Table 2, of the 18 genes, only FBgn0035430 did not fall into classes 9 or 11, which suggests that these important genes in F1 hybrids tend to exhibit lower levels of expression relative to the two parent patterns. Because overexpression of the *tobi* gene can cause growth defects^30^ in *Drosophila melanogaster,* it should be informative to explore the impact of reduced expression levels for *tobi* and its orthologous genes on body weight heterosis.

Two important pathways for drug metabolism, cytochrome P450, and metabolism of xenobiotics by cytochrome P450, were significantly overrepresented by DEGs in the three crosses (Fig. S4). Cytochrome P450 is an ancient gene superfamily originating 3.5 billion years ago^34^, and exists in almost all organisms^35^. Approximately 90 cytochrome P450 genes exist in the *Drosophila melanogaster* genome^36^ and are involved in hormone biosynthesis and metabolism, (e.g., insect juvenile hormone (JH))^37^,^38^,^39^. JH plays an essential role in insect development, metamorphosis, and reproduction^40^,^41^. The heterosis for body weight may therefore be modulated by these pathways. Interestingly, the hybrids in our study showed obvious heterosis for reproduction (data not shown). However, while the JH gene appeared in our DEG analysis, it exhibited an additive pattern in all three cross combinations rather than dominance expression. The potential importance of additive expression gene should therefore not be ignored. Various gene expression patterns, including overdominance, dominance, and additivity, may together contribute to heterosis, even if dominance expression plays the most important role.

Functional analysis placed the 5172 DGEs into 48 subcategories. Genes represented by 41 of the 48 GO terms were overrepresented in all three crosses, suggesting that the same processes were required to manifest body weight heterosis in the different F1 lines (Fig. S3). GO terms representing biological processes, including some related to body weight, such as developmental process, growth, and metabolic process, account for almost half of the 48 categories, suggesting that these biological processes play an important role in heterosis for bodyweight.

GO terms and KEGG pathways unique to each group were observed when dominance DEGs in each group were analyzed separately. For example, cell killing and auxiliary transport protein activity terms were associated only with group 1, while viral reproduction and nutrient reservoir activity were seen only in group 3. The rhythmic process term was associated with both groups 2 and 3 but not with group 1. This suggests that individual groups may manifest specific heteroses affecting processes such as biological rhythm or self-healing, in addition to heteroses that are more generally shared across groups, such as body weight.

The reciprocal cross combinations ♂WT × ♀eyw and ♂eyw × ♀WT were analyzed independently in our experiments and generated distinct expression profiles. The number of DEGs and their classifications also differed (Fig. S2). However, both crosses exhibited heteroses for body weight. This suggests that heterosis can be caused by different genes in different combinations, even in a reciprocal cross. At the same time, these combinations share many genes that also play roles in heterosis. Why reciprocal cross combinations exhibit distinct expression patterns requires further investigation.

Many dominance DEGs identified in our study could be related to the weight trait. Although we also observed heterosis for reproduction related traits in our crosses, GO terms and KEGG pathways related to reproduction were not significantly enriched in the functional analysis of the 215 common DEGs. A possible explanation is that relatively few genes are responsible for the heterosis in this case.

## Conclusion

In summary, our results suggest that: (1) dominance gene expression was the typical expression pattern associated with heteroses in *Drosophila melanogaster*; (2) Various patterns of gene expression including overdominance, dominance, and additivity occur in all the hybrids, and in combination may generate heteroses. However, the molecular mechanisms underlying the differentially expressed genes involved in heterosis are not understood and require further investigation.

## Methods

### Genetic crosses and trait measurement

Three *Drosophila melanogaster* strains of WT (wild-type), eyw (characterized by ebony-body, yellow-body and white-eye) and w1118 (characterized by white-eye) were maintained in tubes and used as the parental lines in this study. w1118 was a gift from the Institute of Genetics and Developmental Biology of the Chinese Academy of Sciences. All *Drosophila melanogaster* lines were maintained on a corn flour, yeast, sugar and agar medium at 24 ± 1 °C^42^. Over a period of eight days, the body weight of each fly was determined within 24 hours after eclosion using an electronic balance with an accuracy of 0.000001 g. Rate of heterosis (RH) was calculated to evaluate heterosis in body weight according to the following equation: RH = [(F1−(P1 + P2)/2)/(P1 + P2)/2] × 100%, where F1, P1 and P2 are the average body-weight of the F1 hybrid and the two parental inbreds, respectively. Statistical analysis was performed following the method of Wu *et al.* (1983)^43^ and the thresholds for significant differences were set at *P* < 0.05 and *P* < 0.01.

### Sample collection and RNA extraction

Twelve samples were collected from the three parental inbred lines and the three F1 hybrids generated by crossing ♂WT × ♀eyw, ♂w1118 × ♀WT and ♂eyw × ♀WT. Two samples (two biological repetitions) were collected for each strain. Each sample consisted of six female and six male flies, 1 day of age. After collection, samples were immediately frozen and stored at −80 °C until RNA extraction. Samples of each strain were designated according to strain and cross as follows: WT_1/WT_2, w1118___1/w1118___2, eyw_1/eyw_2, WTeyw_1/WTeyw_2 (♂WT × ♀eyw), w1118WT_1/w1118WT_2 (♂w1118 × ♀WT), eywWT_1/eywWT_2 (♂eyw × ♀WT).

Total RNA was extracted using TRIZOL® Reagent (Invitrogen, USA) according to the manufacturer’s instructions and then dissolved in DEPC-treated water. RNA concentration was determined using the Qubit® RNA Assay Kit and the Qubit® 2.0 Flurometer (Life Technologies, CA, USA). RNA purity was confirmed using a NanoPhotometer® spectrophotometer (IMPLEN, CA, USA). RNA integrity number (RIN) was assessed using the RNA Nano 6000 Assay Kit and the Bioanalyzer 2100 system (Agilent Technologies, CA, USA). All RNA samples were stored at −80 °C until library construction. The NEBNext® Ultra™ RNA Library Prep Kit for Illumina® (NEB, USA) was used to construct mRNA-seq libraries according to the provided protocol. The mRNA-seq libraries were sequenced on an Illumina Hiseq2000 instrument.

### Read mapping and identification of differentially expressed genes

Reference genome and annotation files were downloaded from the *Drosophila melanogaster* genome website (ftp://ftp.ensembl.org/pub/release-74/fasta/drosophila_melanogaster/dna/; ftp://ftp.ensembl.org/pub/release-74/gtf/drosophila_melanogaster/). High quality reads were acquired from raw reads by excluding reads in which 50% or more bases in the read had quality values (Qphred) lower than 5. TopHat (version 2.0.9)^44^,^45^ was used to align RNA-seq paired-end high quality reads to the reference genome, and HTSeq (version 0.5.4p3)^46^ was used to track read numbers and calculate the RPKM (Reads Per Kilo bases per Million reads) value to estimate the expression level for each gene. Differentially expressed genes between F1 hybrids and the parental lines were identified using the DESeq R package (1.10.1)^47^ with an adjusted P-value < 0.05 (Benjamini-Hochberg [BH] multiple test correction). Gene expression models, including additivity, expression-level dominance (ELD), and transgressive expression, were classified according to Rapp *et al.*^23^ and Li *et al.*^24^, using a Perl script to implement the analysis described by Li *et al.* (2014).

### Functional and pathway analyses using GO and KEGG

Differentially expressed genes were associated with Gene Ontology (GO) terms using the GOseq R package (Release 2.12)^25^ in which the bias caused by differences in gene length was adjusted, and the significant level of the corrected P value was set at less than 0.05 (BH multiple test correction). Background genes are listed in supplementary information (file 2). To identify the metabolic pathways in which the differentially expressed genes were involved, the KEGG (Kyoto Encyclopedia of Genes and Genomes) pathway analysis was also implemented using the KOBAS software package (v2.0)^26^.

### Quantitative polymerase chain reaction and statistical analyses

Total RNA from each of the twelve sequenced samples was reverse-transcribed into first-strand cDNA using the Fast Quant RT Kit (with gDNase) (TIANGEN BIOTECH (Beijing) CO. LTD, China) according to the provided protocol. The expression levels of ten selected genes was determined by real time quantitative PCR (RT-PCR), using SYBR Green I (superReal PreMix Plus, TIANGEN BIOTECH (Beijing) CO. LTD, China) and CFX96 (TM) real-time PCR machines (Bio-Rad Company, USA). 18s rRNA was used as the housekeeping gene standard. Primer sequences for the eleven genes are listed in Table S6. Each 20 μL RT-PCR reaction was carried out in triplicate wells with the following program: 95 °C for 15 min, followed by 40 cycles of 95 °C for 10s, 58 °C for 20 s and 72 °C for 30 s. Relative gene expression was calculated using the delta-delta-Ct method, and differences in expression tested by analysis of variance (ANOVA) using SAS-9.13 (Cary, NC, USA). The thresholds for significant difference were set to P < 0.05 and P < 0.01.

## Additional Information

**How to cite this article**: Wu, X. *et al.* Comparative transcriptome analysis among parental inbred and crosses reveals the role of dominance gene expression in heterosis in *Drosophila melanogaster*. *Sci. Rep.* **6**, 21124; doi: 10.1038/srep21124 (2016).

## Supplementary Material

Supplementary Dataset 1

Supplementary Information

## Figures and Tables

**Table 1:** RH for *Drosophila melanogaster* body weight (μg)^1^.

Hybridized Combination	Body weight of F1 hybrid	Body weight of parents		4MPV	2RH
		Father line(♂)	Mother line(♀)		
♂**WT** × ♀**eyw**	102.66 ± 1.03 (^3^n = 410)	91.49 ± 0.40 (n = 579)	89.59 ± 2.79 (n = 564)	90.54	13.39%**
♂**w1118** × ♀**WT**	109.90 ± 1.06 (n = 445)	104.78 ± 2.61 (n = 514)	91.49 ± 0.40 (n = 579)	98.14	11.98%**
♂**eyw** × ♀**WT**	109.05 ± 2.54 (n = 419)	89.59 ± 2.79 (n = 564)	91.49 ± 0.40 (n = 579)	90.54	20.44%*

^1^Values represent the mean ± SE.

^2^RH: rate of heterosis. Statistical analysis was performed according to Wu *et al.*^43^, * and ** indicate significant difference (*P* < 0.05) and extremely significant difference (*P* < 0.01), respectively.

^3^The number of samples used to determine body weight.

^4^MPV: mid-parent value.

**Table 2:** Dominance expression genes annotated by KEGG pathways and GO.

KEGG pathway	Gene ID	Gene name	Dominance expression type		
			Group 1	Group 2	Group 3
Galactose metabolism pathway (a)/Starch and sucrose metabolism pathway (b)	FBgn0261575 (ab)	*tobi*	9^1^	11	11
	FBgn0035476 (a)	/	9	11	11
	FBgn0002569 (ab)	*Mal-A2*	9	11	11
	FBgn0002570 (ab)	*Mal-A1*	9	11	11
	FBgn0033297 (ab)	*Mal-A8*	9	11	11
	FBgn0033296 (ab)	*Mal-A7*	9	11	11
	FBgn0040252 (b)	*Ugt86Dh*	9	11	11
	FBgn0040250 (b)	*Ugt86Dj*	9	11	11
GO enrichment					
aminoglycan metabolic process	FBgn0030999	*Mur18B*	9	11	11
	FBgn0022700	*Cht4*	9	11	11
	FBgn0036951	/	9	11	11
	FBgn0035430	/	4	2	2
	FBgn0036363	/	9	11	11
	FBgn0013278	*Hsp70Bb*	9	11	11
	FBgn0036203	*Muc68D*	9	11	11
	FBgn0052302	/	9	11	11
	FBgn0036232	/	9	11	11
	FBgn0013279	*Hsp70Bc*	9	11	11

(a) Gene belongs to galactose metabolism pathway. (b) Gene belongs to starch and sucrose metabolism pathway. (ab) Gene belongs to both pathways.

^1^Classification number (cf. Fig. 2) for differential expression pattern.
