# Supplementary material for: Comparative transcriptome analysis among parental inbred and crosses reveals the role of dominance gene expression in heterosis in Drosophila melanogaster
Source: Sci Rep. 2016 Mar 1;6:21124. doi: 10.1038/srep21124 (PMC4772002; doi:10.1038/srep21124)
Supplement: Supplementary Information [file srep21124-s2.doc]

**Comparative transcriptome analysis among parental inbred and crosses reveals the role of dominance gene expression in heterosis in Drosophila melanogaster**

Xianwen Wu^1,2^, Rongni Li^1,2^, Qianqian Li^1,2^, Haigang Bao^1,2*^, Changxin Wu^1,2^

^1^ College of Animal Science and Technology, China Agricultural University, Beijing, China

^2^ National Engineering Laboratory for Animal Breeding, China Agricultural University, Beijing, China

* Correspondence: **Haigang Bao**, Yuanmingyuan West Road NO 2, College Animal Science & Technology, China Agriculture University, Beijing, 100193, P. R. China.

Tel.: +86 010-62734828; fax: +86 010-62734828;

**E-mail: zjbhg@126.com**

**Supplementary Information**

**
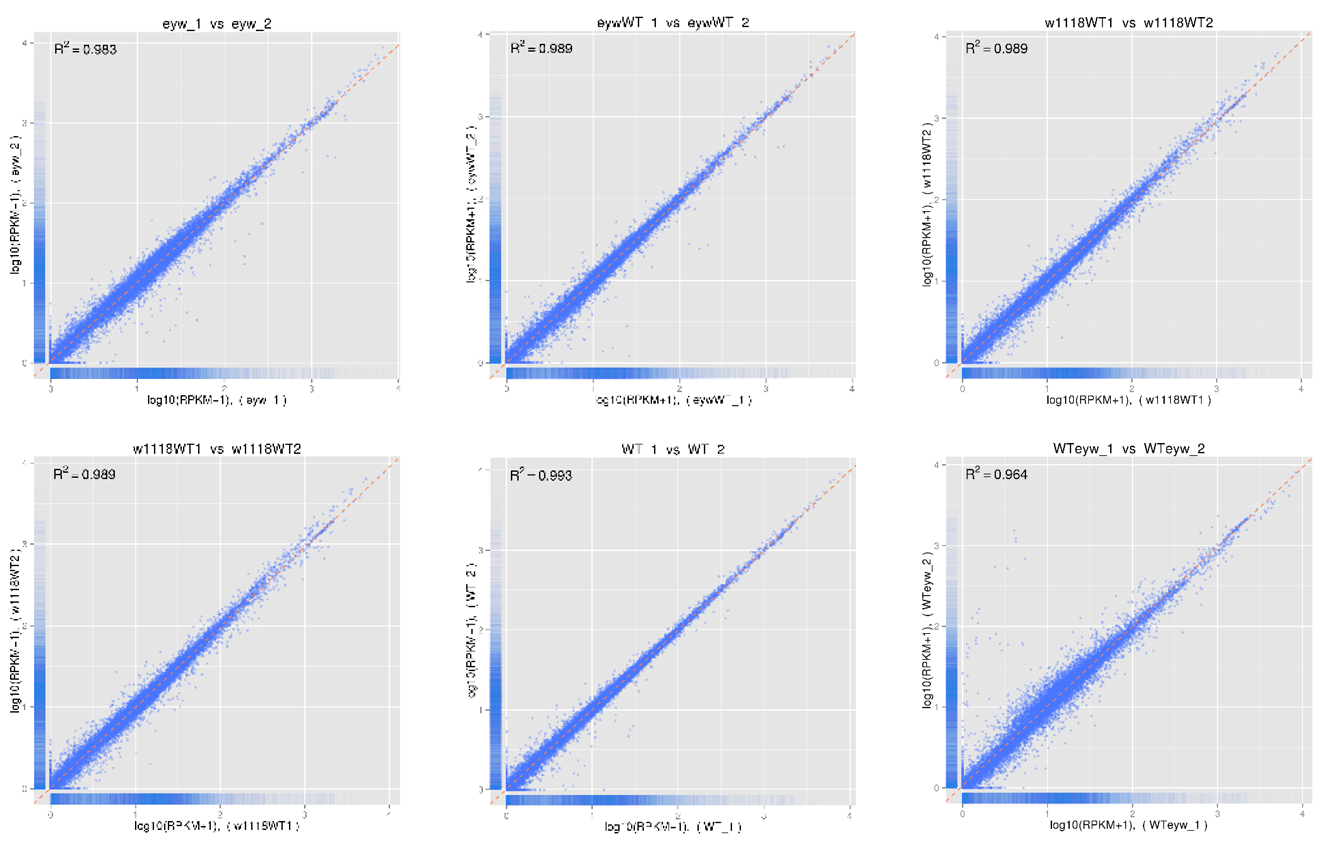
**

**Figure S1．Correlation coefficients (R^2^) between gene expression data sets from two biological replicates in the six groups.**

**
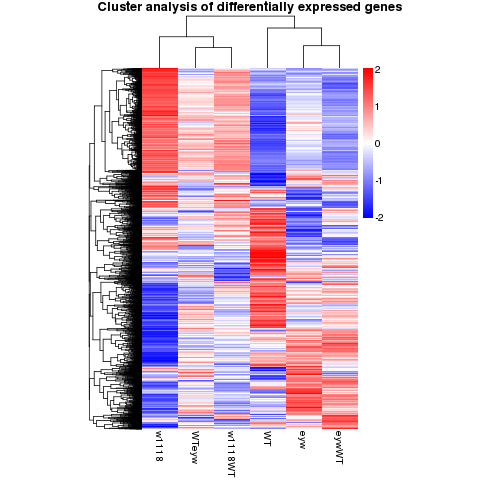
**

**Figure S2．Cluster analysis of differentially expressed genes for the three parental strains and three F1 hybrids.** The red color denotes highly expressed genes, and the blue color denotes genes with lower expression levels. The gradation from red to blue represents the transition from large to small values of log_10_(RPKM+1).

**
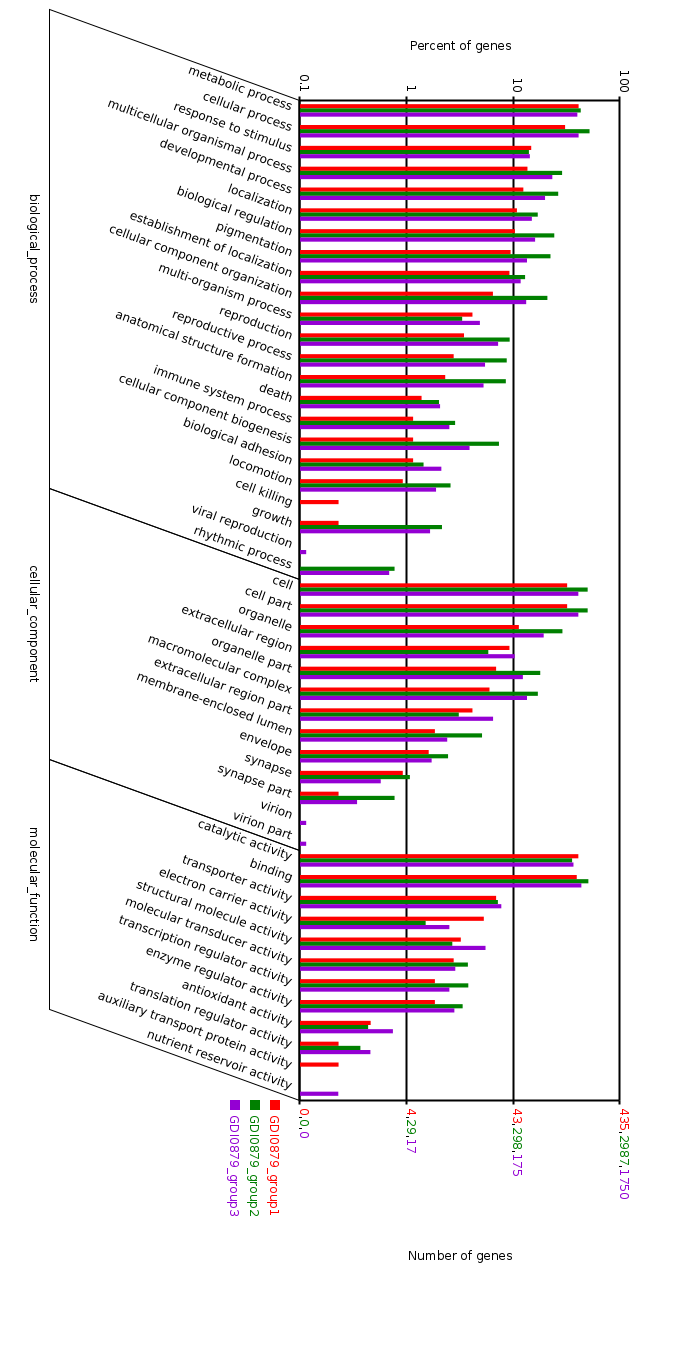
**

**Figure S3．Comparison of Gene Ontology (GO) classifications of dominance DEGs within the three groups.**

**
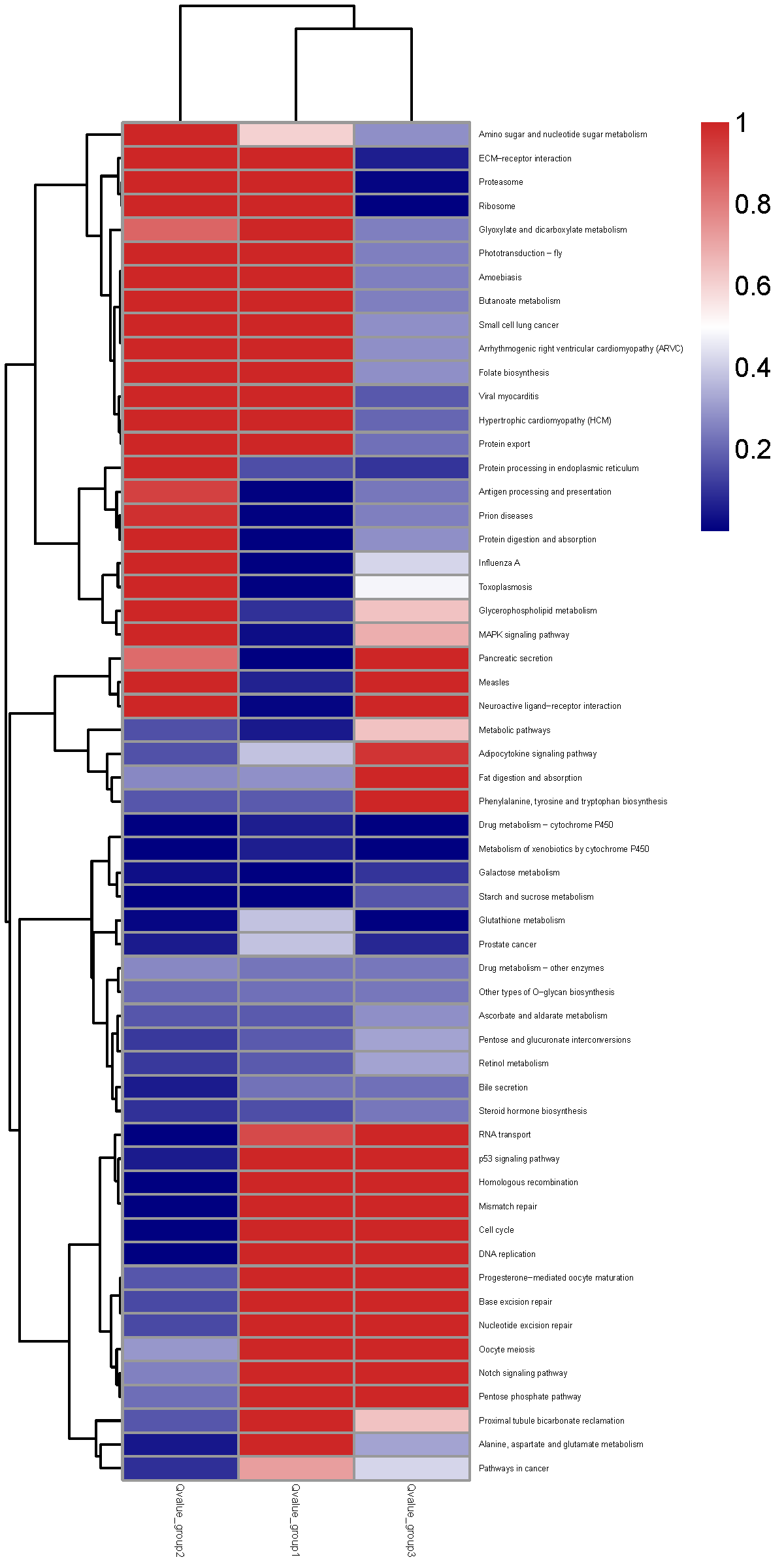
**

**Figure S4．Comparison of KEGG pathway assignments for dominance DGEs within the three groups.**


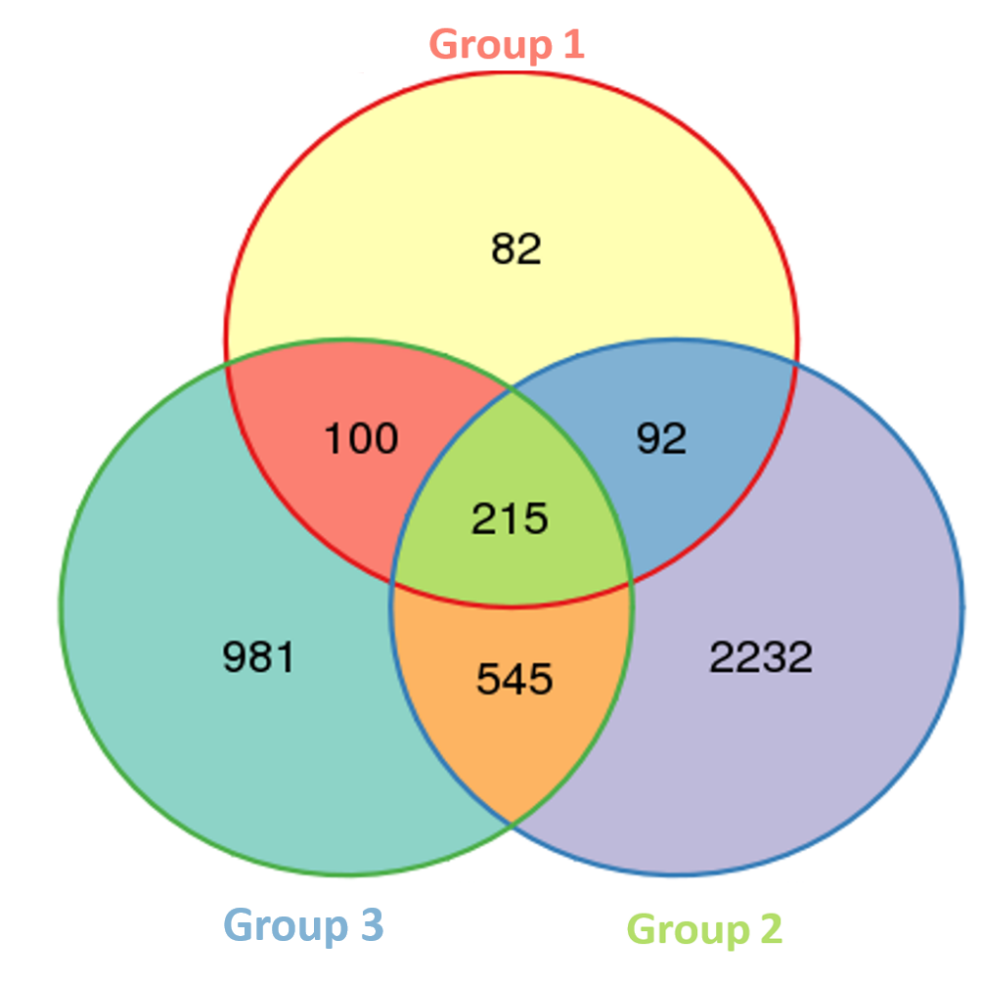
**Figure S5．Numbers of common dominance DEGs within the three groups.** Group 1 shows dominance DEGs between WTeyw and its parents, Group 2 shows dominance DEGs between w1118WT and its parents, and Group 3 shows dominance DEGs between eywWT and its parents.

**
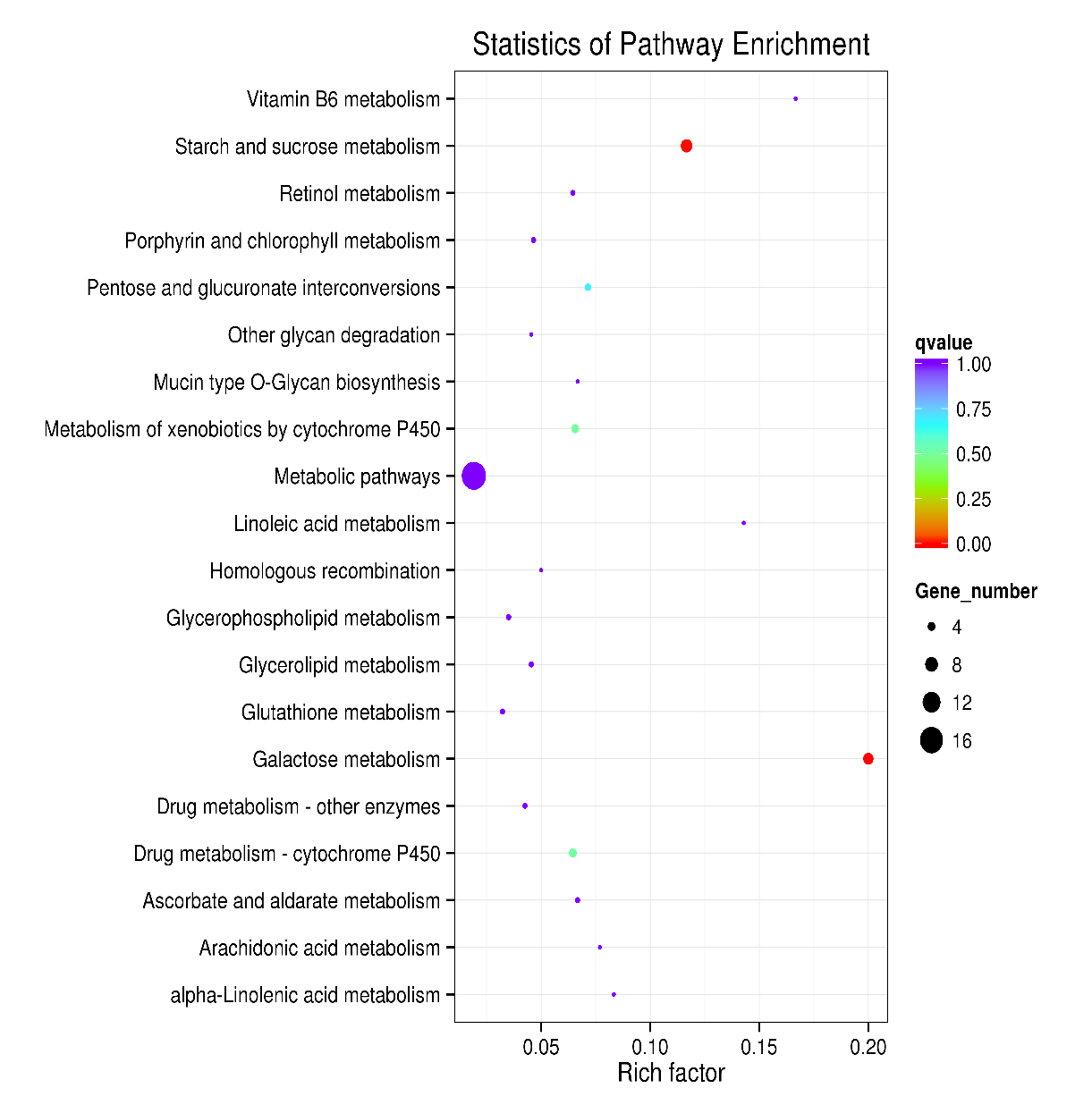
**

**Figure S6． KEGG pathway enrichment amongst the 215 common dominance expressed genes (Table S2).** Enrichment factor = Sample number / Background number.

**
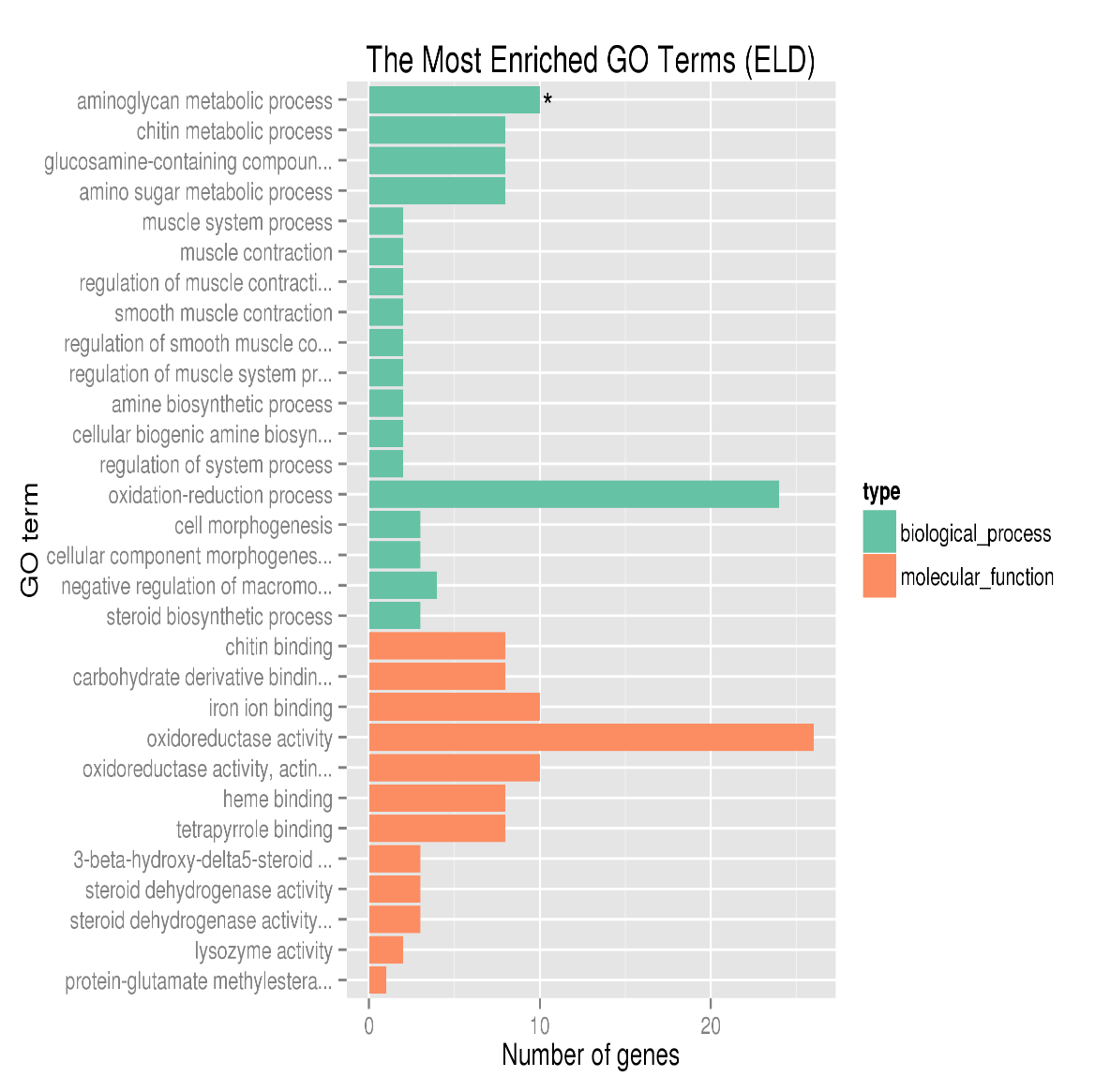
**

**Figure S7． GO enrichment amongst the 215 common dominance expressed genes (Table S3).** GO terms marked with an asterisk (*) were significantly enriched (corrected P-value < 0.05).

**
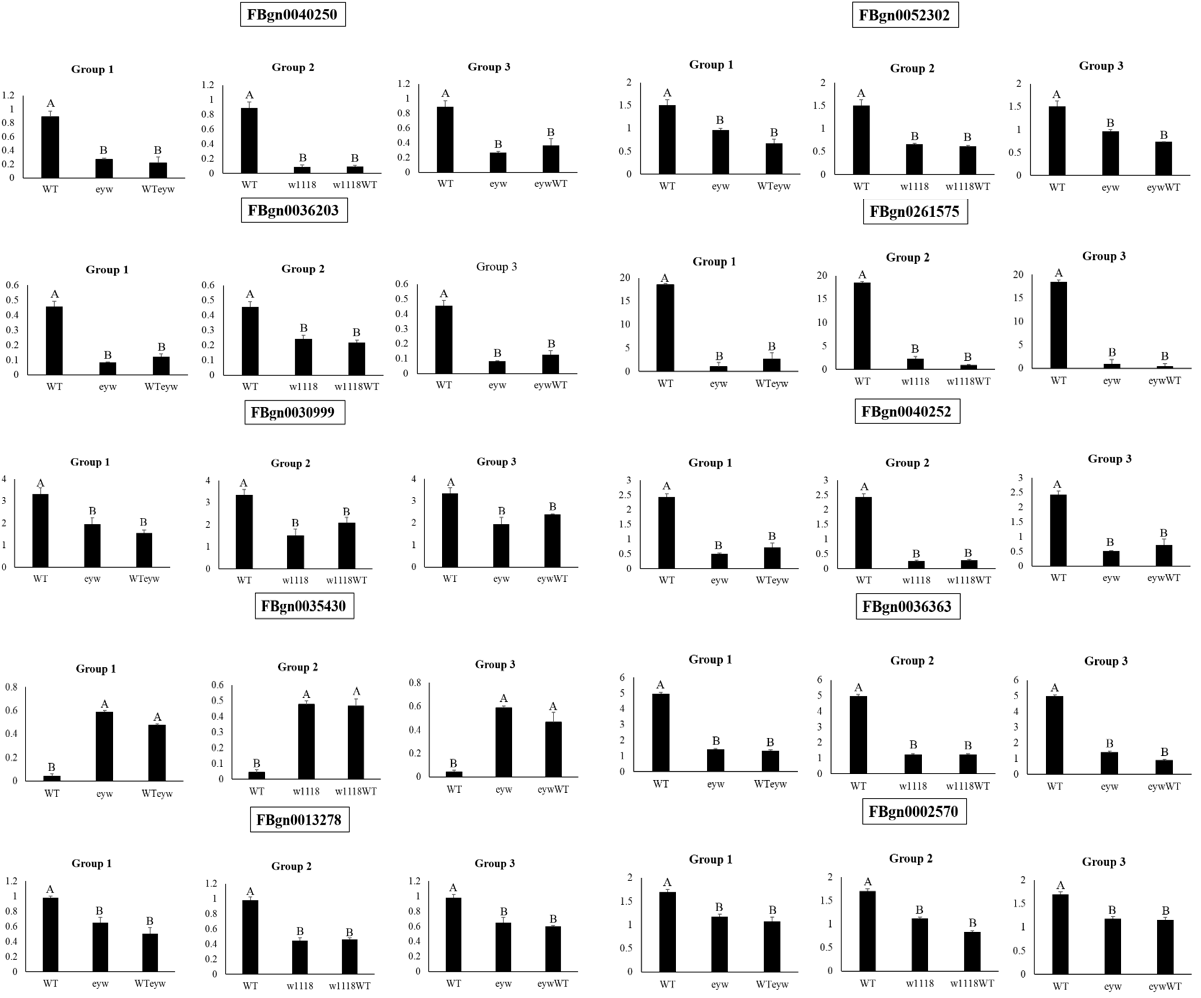
**

**Figure S8. Results of real-time PCR for five genes.** The letters A and B indicate whether values are statistically different (P < 0.05) among the three samples. Identical letters mean that there is no significant difference.

**Table S1. Quality of RNA sequencing data.**

| Sample name | Raw reads | Clean reads | Error  rate (%)^1^ | Q20  (%)^2^ | Q30  (%)^3^ | Number of mapped reads | | | |
| --- | --- | --- | --- | --- | --- | --- | --- | --- | --- |
|  |  |  |  |  |  | Total  (%) | Exon  (%) | Intron  (%) | Intergenic  (%) |
| WT_1 | 59545566 | 54943592 | 0.06 | 94.11 | 86.56 | 83.15 | 98.5 | 0.8 | 0.7 |
| WT_2 | 63954934 | 57755360 | 0.05 | 94.23 | 86.82 | 83.03 | 98.6 | 0.8 | 0.6 |
| WTeyw_1 | 59578184 | 53163132 | 0.06 | 94.01 | 86.39 | 83.38 | 98.2 | 1.1 | 0.7 |
| WTeyw_2 | 63914754 | 57420504 | 0.06 | 94.12 | 86.59 | 83.47 | 98.1 | 1.1 | 0.8 |
| w1118WT_1 | 60714066 | 54808270 | 0.05 | 94.13 | 86.63 | 83.48 | 98.5 | 0.9 | 0.6 |
| w1118WT_2 | 58388088 | 51852734 | 0.06 | 94.08 | 86.54 | 82.32 | 98.3 | 1.0 | 0.7 |
| w1118_1 | 65014390 | 58631070 | 0.06 | 94.23 | 86.65 | 84.03 | 98.7 | 0.7 | 0.6 |
| w1118_2 | 69014422 | 61853798 | 0.06 | 94.22 | 86.64 | 84.08 | 98.6 | 0.8 | 0.6 |
| eywWT_1 | 55046126 | 49980162 | 0.05 | 94.30 | 86.83 | 83.03 | 98.3 | 1.0 | 0.7 |
| eywWT_2 | 58899528 | 52403100 | 0.06 | 94.23 | 86.70 | 83.33 | 98.4 | 1.0 | 0.6 |
| eyw_1 | 63082812 | 56097266 | 0.06 | 94.05 | 86.35 | 82.74 | 98.5 | 0.9 | 0.6 |
| eyw_2 | 58743660 | 53968922 | 0.05 | 94.37 | 87.01 | 83.60 | 98.2 | 1.1 | 0.7 |

^1^Error rate：Calculated by the formula 1 Qphred = –10log_10_ (e), where e is error rate of sequencing.

^2^Q20：The probability of an incorrect base call is ≤ 1%.

^3^Q30：The probability of an incorrect base call is ≤ 0.1%.

| Note: Total differentially expressed genes. Deseq P value with p_adj_ < 0.05 | Total | | eywWT | w1118WT | WTeyw | Categories | | | | **Table S2 Gene expression patterns of DEGs between hybrids and their parents in *Drosophila melanogaster*** |
| --- | --- | --- | --- | --- | --- | --- | --- | --- | --- | --- |
|  | 343 | 123 | 39 | 24 | 60 | 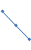 | ♀-F1-♂ | 1 | Additivity |  |
|  |  | 220 | 121 | 87 | 12 | 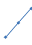 | ♀-F1-♂ | 12 |  |  |
|  | 4706 | 2477 | 907 | 1520 | 50 | 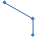 | ♀-F1-♂ | 2 | ELD-♂ |  |
|  |  | 2229 | 751 | 1433 | 45 | 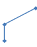 | ♀-F1-♂ | 11 |  |  |
|  | 708 | 314 | 141 | 84 | 89 | 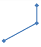 | ♀-F1-♂ | 4 | ELD-♀ |  |
|  |  | 394 | 42 | 47 | 305 | 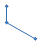 | ♀-F1-♂ | 9 |  |  |
|  | 62 | 8 | 3 | 5 | 0 | 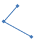 | ♀-F1-♂ | 3 | Transgressive downregulation |  |
|  |  | 41 | 6 | 34 | 1 | 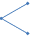 | ♀-F1-♂ | 7 |  |  |
|  |  | 13 | 5 | 8 | 0 | 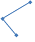 | ♀-F1-♂ | 10 |  |  |
|  | 50 | 1 | 1 | 0 | 0 | 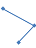 | ♀-F1-♂ | 5 | Transgressive up-regulation |  |
|  |  | 9 | 8 | 1 | 0 | 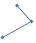 | ♀-F1-♂ | 6 |  |  |
|  |  | 40 | 27 | 12 | 1 | 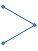 | ♀-F1-♂ | 8 |  |  |
|  | 5869 | | 2051 | 3255 | 563 | Total differentially Expressed genes | | | |  |

**Table S3. Common dominance expression genes amongst the three cross combinations (215 genes)**

| Number | Gene ID | Gene name | Whether associated with body weight trait (√) or unknown (/) |
| --- | --- | --- | --- |
| 1 | FBgn0001230 | Heat shock protein 68 | / |
| 2 | FBgn0001234 | Heat shock RNA ω | / |
| 3 | FBgn0002569 | Maltase A2 | √ |
| 4 | FBgn0002570 | Maltase A1 | √ |
| 5 | FBgn0003714 | / | / |
| 6 | FBgn0004512 | Multi drug resistance 49 | / |
| 7 | FBgn0004575 | Synapsin | √ |
| 8 | FBgn0004777 | Ccp84Ag |  |
| 9 | FBgn0010040 | Glutathione S transferase D4 | √ |
| 10 | FBgn0010395 | Integrin βν subunit | √ |
| 11 | FBgn0010497 | Dietary and metabolic glutamate transporter | / |
| 12 | FBgn0011822 | pepsinogen-like | / |
| 13 | FBgn0013278 | Heat-shock-protein-70Bb | √ |
| 14 | FBgn0013279 | Heat-shock-protein-70Bc | / |
| 15 | FBgn0015038 | Cytochrome P450-9b1 | / |
| 16 | FBgn0015316 | Trypsin 29F | / |
| 17 | FBgn0017558 | Pyruvate dehydrogenase kinase | √ |
| 18 | FBgn0020392 | N-myristoyl transferase | / |
| 19 | FBgn0022700 | Chitinase 4 | / |
| 20 | FBgn0024293 | Serpin 43Ab | / |
| 21 | FBgn0024510 | discs lost | √ |
| 22 | FBgn0025391 | Sarcoglycan δ | √ |
| 23 | FBgn0027929 | Nimrod B1 | / |
| 24 | FBgn0028381 | Death executioner caspase related to Apopain/Yama | / |
| 25 | FBgn0028526 |  | / |
| 26 | FBgn0028950 |  | / |
| 27 | FBgn0028978 | tribbles | √ |
| 28 | FBgn0029826 | / | / |
| 29 | FBgn0030305 | / | / |
| 30 | FBgn0030395 | Meckel syndrome, type 1 homologue | / |
| 31 | FBgn0030484 | Glutathione S transferase T4 | √ |
| 32 | FBgn0030756 | / | / |
| 33 | FBgn0030928 | / | / |
| 34 | FBgn0030930 | UDP-N-acetyl-α-D-galactosamine:polypeptide N-acetylgalactosaminyltransferase 2 | √ |
| 35 | FBgn0030999 | Mucin related 18B | / |
| 36 | FBgn0031004 | / | √ |
| 37 | FBgn0031011 | / | √ |
| 38 | FBgn0031176 | / | √ |
| 39 | FBgn0031306 | / | / |
| 40 | FBgn0031517 | / | √ |
| 41 | FBgn0031522 | / | / |
| 42 | FBgn0031523 | / | / |
| 43 | FBgn0031646 | / | / |
| 44 | FBgn0031695 | Cyp4ac3 | / |
| 45 | FBgn0031703 | / | / |
| 46 | FBgn0032235 | / | / |
| 47 | FBgn0032449 | / | / |
| 48 | FBgn0032663 | / | / |
| 49 | FBgn0032908 | / | / |
| 50 | FBgn0032913 | / | √ |
| 51 | FBgn0033124 | Tetraspanin 42Ec | / |
| 52 | FBgn0033139 | Tetraspanin 42Er | / |
| 53 | FBgn0033170 | secretory Phospholipase A2 | / |
| 54 | FBgn0033188 | Death resistor Adh domain containing target | / |
| 55 | FBgn0033205 | / | √ |
| 56 | FBgn0033296 | Maltase A7 | √ |
| 57 | FBgn0033297 | Maltase A8 | √ |
| 58 | FBgn0033302 | Cyp6a14 | / |
| 59 | FBgn0033304 | Cyp6a13 | √ |
| 60 | FBgn0033464 | / | / |
| 61 | FBgn0033519 | / | / |
| 62 | FBgn0033524 | Cyp49a1 | / |
| 63 | FBgn0033582 | / | / |
| 64 | FBgn0033589 | / | / |
| 65 | FBgn0033592 | / | √ |
| 66 | FBgn0033593 | Listericin | / |
| 67 | FBgn0033698 | / | √ |
| 68 | FBgn0033792 | / | √ |
| 69 | FBgn0033820 | / | / |
| 70 | FBgn0033926 | Activity-regulated cytoskeleton associated protein 1 | / |
| 71 | FBgn0033928 | Arc2 | / |
| 72 | FBgn0033945 | / | / |
| 73 | FBgn0033980 | Cyp6a20 | / |
| 74 | FBgn0033981 | Cyp6a21 | / |
| 75 | FBgn0034133 | / | / |
| 76 | FBgn0034247 | / | / |
| 77 | FBgn0034279 | / | / |
| 78 | FBgn0034328 | Immune induced molecule 23 | / |
| 79 | FBgn0034381 | Lithium-inducible SLC6 transporter | / |
| 80 | FBgn0034480 | / | / |
| 81 | FBgn0034638 | / | √ |
| 82 | FBgn0034657 | Lamin B receptor | √ |
| 83 | FBgn0034664 | / | / |
| 84 | FBgn0034717 | / | / |
| 85 | FBgn0034737 | / | / |
| 86 | FBgn0034858 | eIF2B-δ | / |
| 87 | FBgn0034921 | Decapping protein 1 | √ |
| 88 | FBgn0034935 | Orcokinin | √ |
| 89 | FBgn0035094 | / | / |
| 90 | FBgn0035241 | / | / |
| 91 | FBgn0035290 | debris buster | / |
| 92 | FBgn0035402 | Ubiquitin specific protease 5 | √ |
| 93 | FBgn0035409 | / | / |
| 94 | FBgn0035430 | / | / |
| 95 | FBgn0035476 | / | / |
| 96 | FBgn0035557 | / | / |
| 97 | FBgn0035661 | / | / |
| 98 | FBgn0035904 | Glutathione S transferase O3 | / |
| 99 | FBgn0035921 | / | √ |
| 100 | FBgn0035943 | / | / |
| 101 | FBgn0036196 | / | √ |
| 102 | FBgn0036203 | Mucin 68D | √ |
| 103 | FBgn0036232 | / | / |
| 104 | FBgn0036285 | twin of eyg | / |
| 105 | FBgn0036363 | / | √ |
| 106 | FBgn0036640 | nuclear RNA export factor 2 | / |
| 107 | FBgn0036698 | / | √ |
| 108 | FBgn0036935 | / | / |
| 109 | FBgn0036945 | Snakeskin | √ |
| 110 | FBgn0036951 | / | / |
| 111 | FBgn0037007 | / | / |
| 112 | FBgn0037386 | / | √ |
| 113 | FBgn0037547 | / | / |
| 114 | FBgn0037563 | / | / |
| 115 | FBgn0037580 | Dipeptidyl aminopeptidase III | / |
| 116 | FBgn0037683 | / | / |
| 117 | FBgn0037724 | Frost | / |
| 118 | FBgn0037750 | / | / |
| 119 | FBgn0037761 | / | √ |
| 120 | FBgn0037850 | / | / |
| 121 | FBgn0037870 | / | / |
| 122 | FBgn0037934 | / | / |
| 123 | FBgn0037937 | 48 related 3 | / |
| 124 | FBgn0038257 | Senescence marker protein-30 | √ |
| 125 | FBgn0038299 | Serpin 88Eb | / |
| 126 | FBgn0038347 | Aldehyde oxidase 1 | √ |
| 127 | FBgn0038451 | / | / |
| 128 | FBgn0038461 | / | / |
| 129 | FBgn0038466 | / | / |
| 130 | FBgn0038595 | / | / |
| 131 | FBgn0038640 | / | / |
| 132 | FBgn0038647 | / | / |
| 133 | FBgn0038658 | / | / |
| 134 | FBgn0038680 | Cyp12a5 | √ |
| 135 | FBgn0038717 | / | / |
| 136 | FBgn0038718 | / | / |
| 137 | FBgn0038828 | / | / |
| 138 | FBgn0038865 | / | / |
| 139 | FBgn0039022 | / | / |
| 140 | FBgn0039091 | / | √ |
| 141 | FBgn0039092 | / | √ |
| 142 | FBgn0039232 | sosie | / |
| 143 | FBgn0039310 | / | / |
| 144 | FBgn0039315 | / | / |
| 145 | FBgn0039316 | / | / |
| 146 | FBgn0039420 | / | / |
| 147 | FBgn0039443 | TweedleS | / |
| 148 | FBgn0039470 | / | √ |
| 149 | FBgn0039472 | / | √ |
| 150 | FBgn0039474 | / | √ |
| 151 | FBgn0039736 | / | / |
| 152 | FBgn0039809 | / | / |
| 153 | FBgn0039872 | salty dog | / |
| 154 | FBgn0040104 | lectin-24A | √ |
| 155 | FBgn0040250 | Ugt86Dj | √ |
| 156 | FBgn0040252 | Ugt86Dh | √ |
| 157 | FBgn0040340 | TRAM | / |
| 158 | FBgn0040363 | / | / |
| 159 | FBgn0040705 | NADH dehydrogenase (ubiquinone) B8 subunit | √ |
| 160 | FBgn0040723 | / | / |
| 161 | FBgn0040972 | / | / |
| 162 | FBgn0042110 | / | √ |
| 163 | FBgn0043364 | cabut | √ |
| 164 | FBgn0050360 | Maltase A6 | √ |
| 165 | FBgn0050428 | / | / |
| 166 | FBgn0050456 | / | / |
| 167 | FBgn0051072 | lysosomal enzyme receptor protein | √ |
| 168 | FBgn0051146 | Neuroligin 1 | √ |
| 169 | FBgn0051201 | Glutamate receptor IIE | / |
| 170 | FBgn0051205 | / | / |
| 171 | FBgn0051414 | / | / |
| 172 | FBgn0051562 | / | / |
| 173 | FBgn0051720 | methuselah-like 15 | √ |
| 174 | FBgn0052017 | / | / |
| 175 | FBgn0052195 | / | / |
| 176 | FBgn0052302 | / | √ |
| 177 | FBgn0052557 | / | / |
| 178 | FBgn0052745 | / | / |
| 179 | FBgn0052865 | αγ-element:CR32865 | / |
| 180 | FBgn0053468 | / | √ |
| 181 | FBgn0054026 | / | √ |
| 182 | FBgn0067783 | alternative testis transcripts open reading frame A | √ |
| 183 | FBgn0085205 | / | √ |
| 184 | FBgn0085261 | / | / |
| 185 | FBgn0085302 | / | / |
| 186 | FBgn0085419 | Rad, Gem/Kir family member 2 | √ |
| 187 | FBgn0085484 | Pyridoxal kinase | √ |
| 188 | FBgn0086708 | starvin | √ |
| 189 | FBgn0250815 | Jonah 65Aiv | √ |
| 190 | FBgn0259147 | / | / |
| 191 | FBgn0259918 | inaF-B | √ |
| 192 | FBgn0260767 | / | / |
| 193 | FBgn0261113 | / | √ |
| 194 | FBgn0261560 | Thor | √ |
| 195 | FBgn0261575 | target of brain insulin | √ |
| 196 | FBgn0261625 | / | √ |
| 197 | FBgn0261703 | germ cell-expressed bHLH-PAS | √ |
| 198 | FBgn0261963 | midline | / |
| 199 | FBgn0262536 | / | / |
| 200 | FBgn0262624 | Tetraspan membrane protein in hair cell stereocilia ortholog | √ |
| 201 | FBgn0262993 | / | / |
| 202 | FBgn0263002 | / | / |
| 203 | FBgn0263131 | / | √ |
| 204 | FBgn0263212 | / | / |
| 205 | FBgn0263290 | / | / |
| 206 | FBgn0263593 | Lipin | √ |
| 207 | FBgn0264296 | / | √ |
| 208 | FBgn0264706 | / | / |
| 209 | Novel00011 | / | / |
| 210 | Novel00046 | / | / |
| 211 | Novel00064 | / | / |
| 212 | Novel00076 | / | / |
| 213 | Novel00087 | / | / |
| 214 | Novel00203 | / | / |
| 215 | Novel00352 | / | / |

**Table S4. The 20 most enriched KEGG pathway terms amongst the 215 common dominance DEGs.**

| Number | Term | Sample number | Background number | P-Value | Corrected P-Value |
| --- | --- | --- | --- | --- | --- |
| 1 | Galactose metabolism | 6 | 30 | 6.50E-06 | 0.000753851 |
| 2 | Starch and sucrose metabolism | 7 | 60 | 4.34E-05 | 0.002518818 |
| 3 | Metabolism of xenobiotics by cytochrome P450 | 4 | 61 | 0.016489006 | 0.505003168 |
| 4 | Drug metabolism - cytochrome P450 | 4 | 62 | 0.017413902 | 0.505003168 |
| 5 | Pentose and glucuronate interconversions | 3 | 42 | 0.029738982 | 0.689944376 |
| 6 | Ascorbate and aldarate metabolism | 2 | 30 | 0.083905224 | 1 |
| 7 | Retinol metabolism | 2 | 31 | 0.088780386 | 1 |
| 8 | Vitamin B6 metabolism | 1 | 6 | 0.093164554 | 1 |
| 9 | Linoleic acid metabolism | 1 | 7 | 0.107834955 | 1 |
| 10 | Porphyrin and chlorophyll metabolism | 2 | 43 | 0.152683479 | 1 |
| 11 | Glycerolipid metabolism | 2 | 44 | 0.158357363 | 1 |
| 12 | Drug metabolism - other enzymes | 2 | 47 | 0.175605534 | 1 |
| 13 | alpha-Linolenic acid metabolism | 1 | 12 | 0.177741026 | 1 |
| 14 | Arachidonic acid metabolism | 1 | 13 | 0.191058214 | 1 |
| 15 | Mucin type O-Glycan biosynthesis | 1 | 15 | 0.217056319 | 1 |
| 16 | Glycerophospholipid metabolism | 2 | 57 | 0.234801425 | 1 |
| 17 | Metabolic pathways | 17 | 889 | 0.260729907 | 1 |
| 18 | Glutathione metabolism | 2 | 62 | 0.264894673 | 1 |
| 19 | Homologous recombination | 1 | 20 | 0.278493806 | 1 |
| 20 | Other glycan degradation | 1 | 22 | 0.301712237 | 1 |

**Table S5. The 30 most enriched GO terms amongst the 215 common dominance DEGs.**

| Number | GO accession | Description | Term type | pValue | Corrected pValue | Gene number |
| --- | --- | --- | --- | --- | --- | --- |
| 1 | GO:0006022 | aminoglycan metabolic process | biological_process | 1.26E-05 | 0.049983 | 10 |
| 2 | GO:0008061 | chitin binding | molecular_function | 7.16E-05 | 0.070931 | 8 |
| 3 | GO:0006030 | chitin metabolic process | biological_process | 8.25E-05 | 0.070931 | 8 |
| 4 | GO:1901071 | glucosamine-containing compound metabolic process | biological_process | 8.25E-05 | 0.070931 | 8 |
| 5 | GO:0006040 | amino sugar metabolic process | biological_process | 8.92E-05 | 0.070931 | 8 |
| 6 | GO:0097367 | carbohydrate derivative binding | molecular_function | 0.00014324 | 0.094971 | 8 |
| 7 | GO:0005506 | iron ion binding | molecular_function | 0.00028305 | 0.16085 | 10 |
| 8 | GO:0003012 | muscle system process | biological_process | 0.0014325 | 0.43836 | 2 |
| 9 | GO:0006936 | muscle contraction | biological_process | 0.0014325 | 0.43836 | 2 |
| 10 | GO:0006937 | regulation of muscle contraction | biological_process | 0.0014325 | 0.43836 | 2 |
| 11 | GO:0006939 | smooth muscle contraction | biological_process | 0.0014325 | 0.43836 | 2 |
| 12 | GO:0006940 | regulation of smooth muscle contraction | biological_process | 0.0014325 | 0.43836 | 2 |
| 13 | GO:0090257 | regulation of muscle system process | biological_process | 0.0014325 | 0.43836 | 2 |
| 14 | GO:0016491 | oxidoreductase activity | molecular_function | 0.0016454 | 0.46752 | 26 |
| 15 | GO:0016705 | oxidoreductase activity, acting on paired donors, with incorporation or reduction of molecular oxygen | molecular_function | 0.0018566 | 0.49237 | 10 |
| 16 | GO:0009309 | amine biosynthetic process | biological_process | 0.0022596 | 0.52874 | 2 |
| 17 | GO:0042401 | cellular biogenic amine biosynthetic process | biological_process | 0.0022596 | 0.52874 | 2 |
| 18 | GO:0044057 | regulation of system process | biological_process | 0.0029713 | 0.63322 | 2 |
| 19 | GO:0055114 | oxidation-reduction process | biological_process | 0.0030244 | 0.63322 | 24 |
| 20 | GO:0020037 | heme binding | molecular_function | 0.0034466 | 0.68554 | 8 |
| 21 | GO:0046906 | tetrapyrrole binding | molecular_function | 0.0044275 | 0.8387 | 8 |
| 22 | GO:0000902 | cell morphogenesis | biological_process | 0.011042 | 1 | 3 |
| 23 | GO:0032989 | cellular component morphogenesis | biological_process | 0.011042 | 1 | 3 |
| 24 | GO:0003854 | 3-beta-hydroxy-delta5-steroid dehydrogenase activity | molecular_function | 0.01193 | 1 | 3 |
| 25 | GO:0016229 | steroid dehydrogenase activity | molecular_function | 0.01193 | 1 | 3 |
| 26 | GO:0033764 | steroid dehydrogenase activity, acting on the CH-OH group of donors, NAD or NADP as acceptor | molecular_function | 0.01193 | 1 | 3 |
| 27 | GO:0010605 | negative regulation of macromolecule metabolic process | biological_process | 0.012707 | 1 | 4 |
| 28 | GO:0003796 | lysozyme activity | molecular_function | 0.013826 | 1 | 2 |
| 29 | GO:0006694 | steroid biosynthetic process | biological_process | 0.014031 | 1 | 3 |
| 30 | GO:0008984 | protein-glutamate methylesterase activity | molecular_function | 0.014605 | 1 | 1 |

**Table S6. qPCR Primer sequences**

| **Gene name** | **Gene ID** | **Primer sequences (5'-3')** | **length（bp）** | **Tm** |
| --- | --- | --- | --- | --- |
| ***tobi*** | FBgn0261575 | F:GCTAATGAAGGGCGAGAAG | 327 | 58 |
|  |  | R:ACAGAAAGCGTATGGTGGC |  |  |
| ***Mal-A1*** | FBgn0002570 | F:AGCATCACGGCTCTTCTTC | 127 | 58 |
|  |  | R: TCCGAGTGTCCCTTTCATT |  |  |
| ***Ugt86Dh*** | FBgn0040252 | F:CGAAATCAGCCAGAACATC | 249 | 58 |
|  |  | R:AGAACGGAGTAATCAGCAAGTA |  |  |
| ***/*** | FBgn0052302 | F:AACGGGCTTTACCTTTGATG | 293 | 58 |
|  |  | R:CAGAGCGAGCAGGATTAGACA |  |  |
| ***/*** | FBgn0036363 | F:TATGTTCCCAGCAAGGTTTC | 149 | 58 |
|  |  | R:GCAATAGTAATAGGCATCTCGTC |  |  |
| ***Mur18B*** | FBgn0030999 | F:ACCACATTTACGGTTCGCAAGA | 121 | 58 |
|  |  | R:TCGCAGCACAGGATTCAAGAG |  |  |
| ***/*** | FBgn0035430 | F:TCCATTCGCTCGGACATCA | 268 | 58 |
|  |  | R:CACCAAAGTTGCCATTCAGTTC |  |  |
| ***Hsp70Bb*** | FBgn0013278 | F:CTGGGCACCACCTACTCCT | 123 | 58 |
|  |  | R:CGCCGTCTCCTTCATCTTG |  |  |
| ***Ugt86Dj*** | FBgn0040250 | F:AACGGAGTAATCAGCAAGTAGGA | 238 | 58 |
|  |  | R:CCAGAACATCGGGAGAAGC |  |  |
| ***Muc68D*** | FBgn0036203 | F:CCATCTACAGGAGCGACAG | 178 | 58 |
|  |  | R:TTCCATTCTCAGACCCACT |  |  |
| ***18Sr RNA*** | FBgn0061475 | F:CAGCAGGCGCGTAAATTACC | 133 | 58 |
|  |  | R:TCCTGTATTGTTATTTTTCGTCACTACCT |  |  |

Note: F is forward primer and R is reverse primer.
